# Targeted Delivery of the HLA-B^∗^27-Binding Peptide into the Endoplasmic Reticulum Suppresses the IL-23/IL-17 Axis of Immune Cells in Spondylarthritis

**DOI:** 10.1155/2017/4016802

**Published:** 2017-12-31

**Authors:** Hui-Chun Yu, Kuang-Yung Huang, Ming-Chi Lu, Hsien-Lu Huang, Su-Qin Liu, Ning-Sheng Lai, Hsien-Bin Huang

**Affiliations:** ^1^Department of Medical Research, Dalin Tzu Chi Hospital, Buddhist Tzu Chi Medical Foundation, Chiayi, Taiwan; ^2^Division of Allergy, Immunology, and Rheumatology, Department of Internal Medicine, Buddhist Dalin Tzu Chi Hospital, Chiayi, Taiwan; ^3^School of Medicine, Tzu Chi University, Hualien City, Taiwan; ^4^Department of Life Science and Institute of Molecular Biology, National Chung Cheng University, Chiayi, Taiwan; ^5^Department of Nutrition and Health Science, Fooyin University, Kaohsiung City, Taiwan

## Abstract

Ankylosing spondylitis (AS) is highly associated with the expression of human leukocyte antigen-B27 (HLA-B^∗^27). HLA-B^∗^27 heavy chain (B27-HC) has an intrinsic propensity to fold slowly, leading to the accumulation of the misfolded B27-HC in the endoplasmic reticulum (ER) and formation of the HLA-B^∗^27 HC homodimer, (B27-HC)_2_, by a disulfide linkage at Cys-67. (B27-HC)_2_ displayed on the cell surface can act as a ligand of the killer-cell Ig-like receptor (KIR3DL2). (B27-HC)_2_ binds to KIR3DL2 of NK and Th17 cells and activates both cells, resulting in the activation of the IL-23/IL-17 axis to launch the inflammatory reaction in AS patients. However, activation of the IL-23/IL-17 axis originally derived from the HLA-B^∗^27 misfolding in the ER needs to be characterized. In this study, we delivered two HLA-B^∗^27-binding peptides, KRGILTLKY and SRYWAIRTR, into the ER by using a tat-derived peptide (GRKKRRQRRR)-His_6_-ubiquitin (THU) vehicle. Both peptides are derived from the human actin and nucleoprotein of influenza virus, respectively. Our results demonstrated that targeted delivery of both HLA-B^∗^27-binding peptides into the ER can promote the HLA-B^∗^27 folding, decrease the levels of (B27-HC)_2_, and suppress the activation of the IL-23/IL-17 axis in response to lipopolysaccharide. Our findings can provide a new therapeutic strategy in AS.

## 1. Introduction

Ankylosing spondylitis (AS) is an inflammatory disease that is characterized by inflammatory back pain and asymmetric peripheral oligoarthritis [1–4]. The development of AS is strongly linked with the expression of human leukocyte antigen-B^∗^27 (HLA-B^∗^27) [5, 6]. More than 90% of AS patients express HLA-B^∗^27. HLA-B^∗^27 is one of the major histocompatibility complex (MHC) class I molecules that consist of a heavy chain (*α*-chain) and *β*_2_-microglobulin (*β*_2_m). HLA-B^∗^27 is assembled with an antigenic peptide in the endoplasmic reticulum (ER). The antigenic peptide-bound HLA-B^∗^27 complex is allowed to leave the ER and to be transported to the cell surface for antigen presentation to CD8+ T-cells [7].

The intrinsic propensity of HLA-B^∗^27 heavy chain (B27-HC) allows it to fold slowly in the ER, resulting in the accumulation of misfolded B27-HC and formation of a heavy-chain homodimer, (B27-HC)_2_, which is covalently linked by a disulfide bond at Cys-67 [8–10]. (B27-HC)_2_, displayed on the cell surface, serves as the ligand of the killer-cell Ig-like receptor (KIR3DL2) [11, 12]. KIR3DL2 is present on the membranes of natural killer (NK) cells and T-helper 17 (Th17) cells. Recent studies have demonstrated that (B27-HC)_2_ can interact with the KIR3DL2 receptor and promote the survival and growth of both NK cells and Th17 cells [13, 14]. Activation of NK cells and Th17 cells by (B27-HC)_2_ may make one of the contributions to the pathogenesis of AS.

In addition, the misfolded B27-HC accumulated in the lumen of the ER can associate with Grp78/Bip that functions as a chaperone protein [1, 15, 16]. Under normal conditions without remarkable accumulation of misfolded and unfolded proteins in the lumen of the ER, Grp78/Bip binds to three sensors, ATF6, IRE1, and PERK, of unfolded protein response (UPR) on the membrane of the ER and blocks their functions [17, 18]. The buildup of misfolded B27-HC in the ER provides the new target for Grp78/Bip binding. Consequently, Grp78/Bip discharges from its initial complexes, allowing the sensor proteins to induce UPR [17]. Indeed, the misfolded B27-HC overexpressed in HMy2.C1R cells was observed to associate with Grp78/Bip in the coimmunoprecipitation assay [15, 16]. In the HLA-B^∗^27 transgenic rat, the ER stress induced by the overexpressed HLA-B^∗^27 activates the UPR, upregulates the activation of NF-*κ*B, and promotes the expression of proinflammatory cytokines such as tumor necrosis factor-*α* (TNF-*α*), IL-1, IL-6, and IL-23 [19–21]. Bone marrow-derived macrophages releasing IL-23 to activate Th17 cells were observed when UPR was induced by the accumulation of misfolded B27-HC in transgenic rats.

Recent studies have indicated that the HLA-B^∗^27-binding peptide in the ER can promote HLA-B^∗^27 folding and suppress the formation of (B27-HC)_2_ [22, 23]. In our previous studies, we have designed a novel His_6_-ubiquitin-tagged Tat-derived peptide (THU) vehicle to deliver the HLA-B^∗^27-binding peptides, RRFKEGGRGGKY and RRYLENGKETL, from the extracellular space into the ER [16]. Both delivered peptides are derived from the DNA primase of *Chlamydia trachomatis* and human B27-HC, respectively [24, 25]. The primary sequence of THU, from the N-terminus to the C-terminus, contains a Tat-derived peptide, a His_6_ tag, and ubiquitin. The cargo peptide is immediately linked to the C-terminus of ubiquitin. The human immunodeficiency virus Tat-derived peptide, GRKKRRQRRR, is a small basic peptide that can efficiently translocate various types of cargo, including oligopeptides, across membranes [26, 27]. The THU-HLA-B^∗^27-binding peptide fusion protein was rapidly translocated into the cytosol, where the HLA-B^∗^27-binding peptide was discharged from THU by a specific cleavage reaction carried out by cytosolic ubiquitin C-terminal hydrolases (UCHs). The released peptide was then translocated into the lumen of the ER by the transporter associated with antigen processing (TAP) [28, 29]. In the ER, the HLA-B^∗^27-binding peptide can promote the folding of B27-HC and suppress the formation of (B27-HC)_2_. The levels of (B27-HC)_2_ were decreased, and the concentrations of the assembled HLA-B^∗^27 HC/*β*_2_m/peptide complex on the cell membrane were increased. However, it remained unknown whether promotion of HLA-B^∗^27 folding in the ER can suppress the IL-23/IL-17 axis of the immune cells in AS. In this study, we delivered two new HLA-B^∗^27-binding peptides, KRGILTLKY and SRYWAIRTR, into the ER by the THU vehicle. Our results demonstrated that targeted delivery of the HLA-B^∗^27-binding peptide into the ER can promote the HLA-B^∗^27 folding and suppress the IL-17A or IL-17A/IL-23 expressions of PBMCs isolated from AS patients in response to lipopolysaccharide (LPS), indicating the strong linkage between HLA-B^∗^27 misfolding in the ER and activation of the IL-23/IL-17 axis in AS.

## 2. Materials and Methods

Acrylamide, nickel chloride, dithiothreitol (DTT), Tris, Luria-Bertani (LB) broth, ampicillin, EDTA, imidazole, isopropyl *β*-D-1-thiogalactopyranoside (IPTG), phenylmethylsulfonyl fluoride (PMSF), lipopolysaccharide (LPS), MOPS, puromycin, benzamidine, sodium dodecyl sulfate, sodium azide, sodium chloride, TEMED, ammonium persulfate, glycine, and hygromycin B were obtained from Sigma-Aldrich (St. Louis, MO, USA). Chelating Sepharose and SP Sepharose were purchased from GE Healthcare (Uppsala, Sweden). Preparations of tat-derived peptide (GRKKRRQRRR)-His_6_-ubiquitin (THU) and THU-RRFKEGGRGGKY (THUC) followed the methods [16].

### 2.1. Protein Purification

The cDNAs encoding THU-KRGILTLKY (THUA), THU-SRYWAIRTR (THUNP), His_6_-ubiquitin-KRGILTLKY (HUA), and His_6_-ubiquitin-SRYWAIRTR (HUNP) were constructed by a two-step polymerase chain reaction (PCR). The primers used for PCR amplification are listed in Supplementary Figure S1. The resulting product from the first PCR was used as a template for the second PCR. The resulting product of the second PCR was cloned into pET28a at the *Nhe*I/*Xho*I sites of the plasmid. *E. coli* BL21 (DE3) cells transformed with the recombinant vector encoding THUA, THUNP, HUA, or HUNP were grown in one liter of LB broth with 0.3 g/liter kanamycin sulfate at 37°C with shaking at 250 rpm. When the absorbance at 600 nm was between 0.6 and 1.0, 0.38 g of IPTG was added for a final concentration of 1 mM to induce protein expression. Bacteria were harvested by centrifugation after three-hour induction. The pelleted cells were resuspended in 30 ml of 20 mM Tris-HCl buffer (pH 7.9), containing 0.5 M NaCl, 0.2 mM PMSF, 0.02% sodium azide, and 4 mM benzamidine, and lysed by French press. The insoluble components were removed by centrifugation at 20,000 g for 20 min. The supernatant was loaded onto a Ni^2+^ Sepharose column (2.5 × 10 cm). After washing with one volume of the same buffer, bound proteins were eluted with a linear imidazole gradient from 5 mM (500 ml) to 1.0 M imidazole (500 ml) in the same buffer. The fractions containing the expressed protein were pooled, dialyzed against the deionized water (two liters) with five changes during 36 hours to remove the excess reagents, and lyophilized to powder. The lyophilized protein was then dissolved with 20 ml of 20 mM MOPS (pH 7.0) and 0.2 mM EDTA. All components were resolved by SP Sepharose chromatography (2.5 × 20 cm) with a linear gradient from 0 (500 ml) to 2 M NaCl (500 ml). The fractions containing the target protein were pooled, dialyzed against the deionized water, and lyophilized.

### 2.2. Ethics Statement

Patients defined according to the modified New York criteria [30] were recruited into the study between January 2014 and December 2014 in a regional teaching hospital in Southern Taiwan. The experimental procedures for the separation of human PMBCs from AS patients have been evaluated and approved by the Institutional Review Board (IRB) of Dalin Tzu Chi Hospital, Buddhist Tzu Chi Medical Foundation, Taiwan (number B10302005). Written informed consent was obtained from all study patients. Human PBMCs from the AS patients were prepared following the methods as previously described [31].

### 2.3. Western Blotting Analysis

HMy2.C1R cells (ATCC, Manassas, VA) belong to the B-lymphoblast cells that deficiently express the HLA-A^∗^ or HLA-B^∗^ genes [32]. Both C1R-B^∗^27:04 cells and TAP1-knockdown C1R-B^∗^27:04 cells were derived from HMy2.C1R cells that stably overexpress HLA-B^∗^27:04 heavy chain (B2704-HC), a subtype of B27-HC [16]. C1R-B^∗^27:04 cells (3 × 10^6^ cells/well) were grown on 24-well plates and maintained in 1 ml of Iscove's modified Dulbecco's medium (IMDM) (Invitrogen, Carlsbad, CA) with 10% fetal bovine serum (FBS) (Invitrogen) and 200 *μ*g/ml hygromycin B. C1R-B^∗^27:04 cells (3 × 10^6^ cells/well) with TAP1 knockdown were maintained in the same medium with 0.4 *μ*g/ml puromycin. Cells were treated with 20 *μ*g of THUA, THU, HUA, THUNP, or HUNP and harvested at the indicated time. Membrane proteins were extracted using a ProteoExtract Native Membrane Protein Extraction Kit (Calbiochem, Darmstadt, Germany) and following the manufacturer's instruction. Fresh iodoacetamide (10 mM) was included in all buffers to block disulfide bridge formation during membrane protein extraction. Aliquots of extracted membrane proteins in the supernatant were separated by nonreducing SDS-PAGE (10%); transferred to polyvinyl difluoride membranes (GE Healthcare); immunoblotted with BH2, an anti-misfolded HLA-B^∗^27 monoclonal antibody [16]; and analyzed by horseradish peroxidase- (HRP-) conjugated goat anti-mouse IgG (Santa Cruz Biotechnology, Dallas, TX).

### 2.4. Flow Cytometry

All assays followed the methods as described [16]. C1R-B^∗^27:04 cells (2 × 10^6^ cells per well) or TAP1-knockdown C1R-B^∗^27:04 cells (2 × 10^6^ cells per well) were grown in 1 ml IMDM with 10% FBS, 200 *μ*g/ml hygromycin B, and 0.4 *μ*g/ml puromycin. Cells were treated with 10 *μ*M THUA, 10 *μ*M THUNP, 10 *μ*M THU, 10 *μ*M HUA, or 10 *μ*M HUNP overnight, followed by washing with PBS three times and staining with a W6/32 antibody (Abcam, Cambridge, MA; 1 : 500 dilution) in the dark for 30 min. After washing with PBS three times, the stained cells were incubated with goat FITC-conjugated anti-mouse IgG (Millipore, Temecula, CA; 1 : 500 dilution) in the dark for 30 min, washed with PBS three times, and analyzed by flow cytometry.

### 2.5. Quantitative Real-Time Reverse Transcriptase PCR

PBMCs (3 × 10^6^ cells) isolated from AS patients were maintained in RPMI1640 medium and treated with 200 ng LPS plus 20 *μ*g THUC, THUA, or THUNP at 37°C with 5% CO_2_ for 24 h. The total RNAs of treated PBMCs were isolated by using the QIAamp RNA Blood Mini Kit (QIAGEN GmbH, Germany). IFN-*γ*, TNF-*α*, IL-6, IL-17A, or IL-23 mRNA was amplified by real-time PCR using a One Step SYBR Ex Taq qRT-PCR kit (TaKaRa, Shiga, Japan).

### 2.6. Statistical Analysis

Data are displayed as means ± SD. *P* values were obtained by the Mann–Whitney *U* test. *P* values less than 0.05 were considered statistically significant.

## 3. Results

### 3.1. Protein Purification

**Figure 1:**
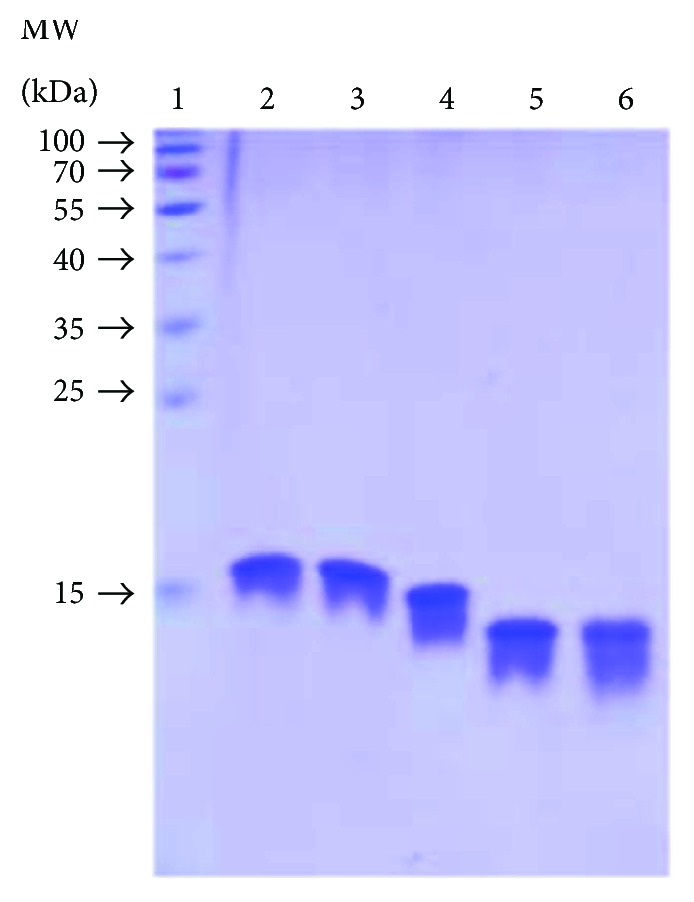
SDS-PAGE analysis of the recombinant THUNP, THUA, THU, HUNP, and HUA. An aliquot (2 *μ*g) of the recombinant THUNP, THUA, THU, HUNP, and HUA was resolved by SDS-PAGE (15%) and stained with Coomassie Brilliant Blue. Lane 1: molecular weight marker, lane 2: THUNP, lane 3: THUA, lane 4: THU, lane 5: HUNP, and lane 6: HUA.

The recombinant THU-KRGILTLKY (THUA), THU-SRYWAIRTR (THUNP), His_6_-ubiquitin-KRGILTLKY (HUA), or His_6_-ubiquitin-SRYWAIRTR (HUNP) was individually overexpressed in *E. coli* BL21 (ED3) and purified by a two-step chromatography. Both KRGILTLKY and SRYWAIRTR peptides are derived from the human actin and nucleoprotein of influenza virus, respectively [33]. After rupture of cells by French press, the crude extract was purified by Ni^2+^ Sepharose chromatography and SP Sepharose chromatography. The homogeneity of each purified protein was analyzed by SDS-PAGE (Figure 1).

### 3.2. Targeted Delivery of HLA-B^∗^27-Binding Peptides into the ER Promotes HLA-B^∗^27 Folding

**Figure 2:**
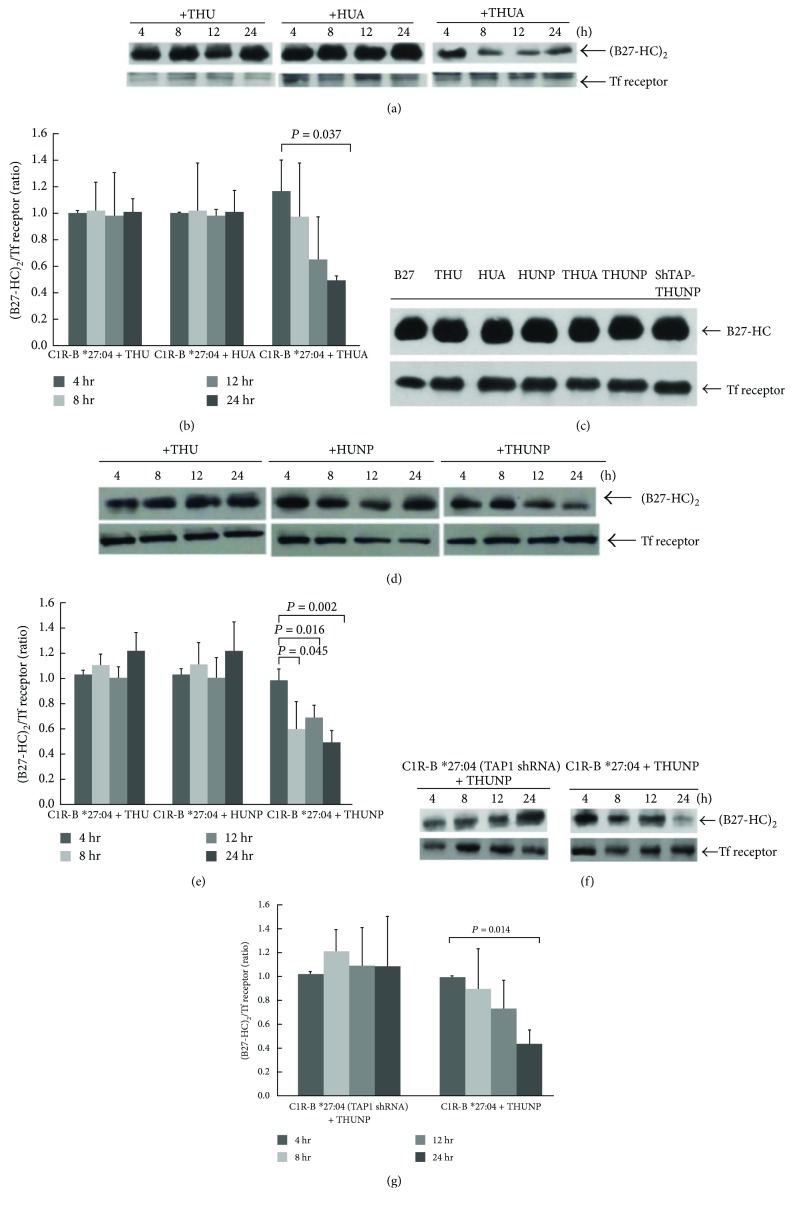
The effect of treatment with THUA or THUNP on the (B27-HC)_2_ production of C1R-B^∗^27:04 cells. After C1R-B^∗^27:04 cells were treated with each recombinant protein for the indicated time, membrane proteins were extracted. 50 *μ*g of extract was analyzed by nonreducing SDS-PAGE (10%) and immunoblotted with a BH2 monoclonal antibody and anti-transferrin (Tf) receptor antibody. Tf receptor acts as an internal control. (a) THUA treatment for 12 h, but not THU or HUA treatment, significantly decreased the levels of (B27-HC)_2_. (b) The ratio of (B27-HC)_2_/Tf receptor averaged from three independent experiments in (a) is plotted against the time of THU, HUA, or THUA treatment (mean ± SD, *n* = 3). (c) Treatment of C1R-B^∗^27:04 cells with THU, HUA, HUNP, THUA, THUNP, or TAP-1 knockdown does not affect the expression levels of B27-HC. C1R-B^∗^27:04 cells were treated with 20 *μ*g of THU, HUA, HUNP, THUA, or THUNP for 24 h. The membrane proteins were extracted. An aliquot (50 *μ*g) of the extracted protein was analyzed by reducing SDS-PAGE and immunoblotted with the BH2 monoclonal antibody. (d) Treatment with the THUNP reduces the formation of (B27-HC)_2_. (e) The ratio of (B27-HC)_2_/Tf receptor averaged from three independent experiments in (d) is plotted against the time of THU, HUNP, or THUNP treatment (mean ± SD, *n* = 3). (f) TAP1-knockdown suppresses the THUNP-induced reduction of (B27-HC)_2_. (g) The ratio of (B27-HC)_2_/Tf receptor averaged from three independent experiments in (f) is plotted against the time of THUNP treatment (mean ± SD, *n* = 3). *P* values were obtained by the Mann–Whitney *U* test.

We used the THU vehicle to deliver two HLA-B^∗^27-binding peptides, KRGILTLKY and SRYWAIRTR, to the ER of C1R-B^∗^27:04. We firstly verified whether treatment with THUA lowered the production of (B27-HC)_2_ in C1R-B^∗^27:04 cells. BH2, a monoclonal antibody that can recognize (B27-HC)_2_, was used in our early study [16, 34]. Figures 2(a) and 2(b) show that the levels of (B27-HC)_2_ are decreased in THUA-treated cells, whereas neither THU nor HUA treatment changes the levels of (B27-HC)_2_. Treatment of C1R-B^∗^27:04 with THUA, HUA, THUNP, HUNP, or THU had no effect on the expression levels of B27-HC (Figure 2(c)). Thus, it is clear that THUA enters the cells via the Tat peptide. The cargo peptide is liberated from THUA in the cytosol via cleavage by endogenous ubiquitin C-terminal hydrolases and then is transported into the lumen of the ER by TAP to promote B27-HC folding. A similar consequence was obtained when C1R-B^∗^27:04 cells were treated with THUNP. The formation of (B27-HC)_2_ in C1R-B^∗^27:04 cells was significantly suppressed by the treatment with THUNP (Figures 2(d) and 2(e)). Neither HUNP nor THU treatment was capable of suppressing the production of (B27-HC)_2_ (Figures 2(d) and 2(e)). TAP, a heterodimeric protein consisting of TAP1 and TAP2 subunits, is located on the ER membrane, belonging to one of the ATP-binding cassette (ABC) transporter members [28]. The cytosolic peptides are translocated by TAP into the ER, where they assemble with MHC class I molecules for shipping to the cell surface. We demonstrated that knockdown of a TAP1 subunit can disrupt the THUNP-promoted reduction of (B27-HC)_2_ formation (Figures 2(f) and 2(g)). However, knockdown of TAP1 did not affect the expression of B27-HC in C1R-B^∗^27:04 cells (Figure 2(c)), suggesting that suppression of (B27-HC)_2_ formation by THUNP treatment was due to the impairment of TAP activity.

### 3.3. The Delivered Peptides Can Be Displayed on the Cell Surface

**Figure 3:**
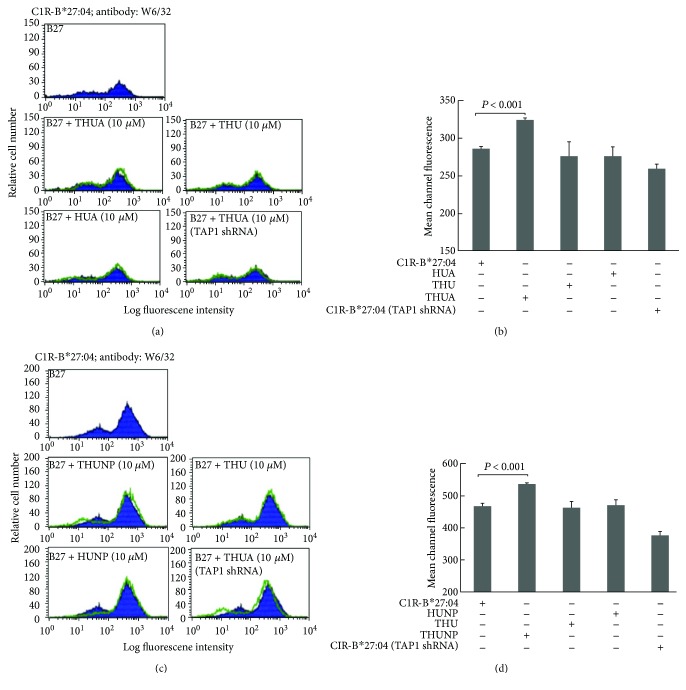
Treatment with either THUA or THUNP increases the antigenic peptide targeted to the cell surface of C1R-B^∗^27:04. (a) Treatment with THUA, but not with THU or HUA, increases the HLA-B^∗^27:04 HC/*β*_2_m/KRGILTLKY complex presented at the cell surface of C1R-B^∗^27:04, as detected by flow cytometry. Knockdown of TAP1 ruins the antigenic peptide, KRGILTLKY, presented on the cell surface. (b) Mean channel fluorescence measured in (a) was averaged from three independent experiments (mean ± SD, *n* = 3). Values of mean channel fluorescence measured from the large population of C1R-B^∗^27:04 cells that display the high levels of HLA-B^∗^27:04 on the cell surface were increased when cells were treated with THUA. (c) Treatment with THUNP, but not with THU or HUNP, increases the HLA-B^∗^27:04 HC/*β*_2_m/SRYWAIRTR complex presented at the cell surface, as detected by flow cytometry. TAP1 knockdown impairs the antigenic peptide, SRYWAIRTR, presented on the cell surface. (d) Mean channel fluorescence measured in (c) is increased when C1R-B^∗^27:04 cells were treated with THUNP. Values (mean ± SD, *n* = 3) measured from the high population of cells that display the high levels of HLA-B^∗^27:04 on the cell surface are averaged from three independent experiments. Statistical significance was obtained by the Mann–Whitney *U* test.

Assuming that the actin peptide or NP peptide has been targeted to the ER lumen of C1R-B^∗^27:04 by the THU vehicle, it will enhance B27-HC folding and assemble into the B27-HC/*β*_2_m heterodimeric complex. Thus, the amount of the assembled B27-HC/*β*_2_m/peptide heterotrimeric complex will be increased and then will be transported to the cell surface. Two populations of C1R-B^∗^27:04 cells were observed by flow cytometric analysis (Figures 3(a) and 3(c)). Cells in the small population account for around 22% of the total C1R-B^∗^27:04 cells but display the low levels of HLA-B^∗^27:04 on the cell surface, as judged by the W6/32 staining. This may suggest that the expression of the B27-HC is low in this population. Another population contains most of the C1R-B^∗^27:04 cells. The cells in this group display the high levels of the HLA-B^∗^27:04 on the cell surface. As a result, the levels of the assembled B27-HC/*β*_2_m/peptide heterotrimeric complex on the cell membrane will be increased. Thus, we analyzed whether the ER-targeted actin or NP peptide has been transported to the cell plasma membrane by the B27-HC/*β*_2_m complex of C1R-B^∗^27:04. If the folded B27-HC/*β*_2_m/peptide heterotrimeric complex is present on the plasma membrane, it will be identified by W6/32, a monoclonal antibody that recognizes the folded MHC class I molecules [35]. After C1R-B^∗^27:04 cells were treated with THUA or THUNP, the population of W6/32-reactive cells was clearly increased (Figures 3(a)–3(d)), suggesting that the actin peptide or NP peptide has been presented on the cell surface by the B27-HC/*β*_2_m complex. However, an increased W6/32-reactive cell population was not observed when C1R-B^∗^27:04 cells were treated with THU, HUA, or HUNP (Figures 3(a)–3(d)). Moreover, impairment of TAP function by knockdown of TAP1 diminished the translocation of the antigenic peptide into the ER, resulting in lowering the assembly of the B27-HC/*β*_2_m complex with the antigenic peptide and reducing the peptide presentation on the cell membrane. Apparently, the cargo peptide presented on the cell surface of TAP-knockdown cells was suppressed even though they were treated with THUA or THUNP (Figures 3(a)–3(d)).

### 3.4. Targeted Delivery of HLA-B^∗^27-Binding Peptides into the ER Suppresses the IL-23/IL-17 Axis of PBMCs Isolated from AS Patients in Response to LPS

**Figure 4:**
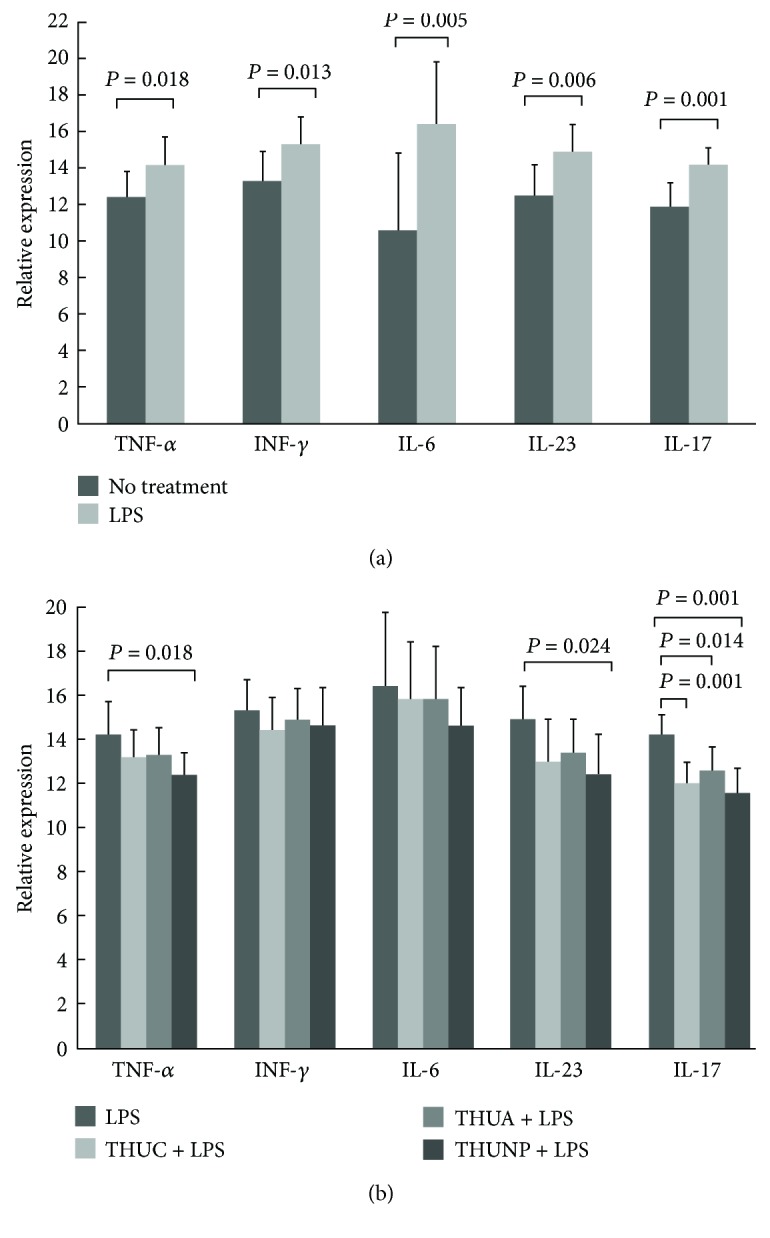
The effects of treatment with THUC, THUA, or THUNP on the production of proinflammatory cytokine mRNAs in PBMCs isolated from AS patients in response to LPS. The suitable primers were synthesized as described [40]. Relative expression levels of mRNA were defined by the following equation: (40 − threshold cycle [Ct] after adjustment by the expression of 18S rRNA). (a) Treatment with LPS promotes the mRNA expression of cytokines, TNF-*α*, INF-*γ*, IL-6, IL-23, and IL-17A mRNAs (mean ± SD, *n* = 9). *P* values were obtained by *t*-test. (b) Treatment with THUC, THUA, or THUNP reduced the expression of IL-17A mRNA. Treatment with THUNP also suppressed the expression of TNF-*α* and IL-23 mRNAs (mean ± SD, *n* = 9). *P* values were obtained by one-way analysis of variance test followed by Sidak multiple comparison tests.

Several lines of evidence have indicated that the IL-23/Th17 axis is highly involved in AS pathogenesis [21, 36–38]. Then, we asked whether targeted delivery of the HLA-B^∗^27-binding peptide to the ER can suppress the IL-23/IL-17 expression of PBMCs isolated from AS patients in response to LPS. Cytokine expression in the HLA-B^∗^27-expressing PBMCs was stimulated by LPS in the absence of THUA, THUC, or THUNP (Figure 4(a)).  Figure 4(b) shows that targeted delivery of the HLA-B^∗^27-binding peptide to the ER by THU can suppress the expression of IL-17A by HLA-B^∗^27-expressing PBMCs. We also observed that treatment with THUNP can reduce the IL-23 and TNF-*α* expressions of PBMCs (Figure 4(b)). Targeted delivery of the HLA-B^∗^27-binding peptide into the ER failed to affect the expression of IL-6 (Figure 4(b)) and IFN-*γ* mRNAs (Figure 4(b)).

## 4. Discussion

An overwhelming expression of B27-HC has been proved to spontaneously develop the AS-like disease in the transgenic rat [19–21]. In the HLA-B^∗^27 transgenic rat model, accumulation of the misfolded B27-HC in the ER can trigger UPR. The levels of mRNAs for UPR markers, including Grp78, CHOP, and XBP-1 transcription factor, were significantly increased in bone marrow-derived macrophages, where accumulation of misfolded heavy chain induced by HLA-B^∗^27 upregulation highly correlated with the magnitude of the UPR. IL-23 production in bone marrow-derived macrophages suffering a UPR triggered by thapsigargin or by HLA-B^∗^27 misfolding was synergistically stimulated by LPS. IL-23 is a key cytokine to induce the differentiation of naïve CD4+ T-cells into Th17 cells that produce proinflammatory cytokines, including IL-17 [39]. Stimulation of the IL-23/IL-17 axis provides the strong linkage between HLA-B^∗^27 misfolding and UPR in transgenic rats. However, the linkage between activation of UPR and production of proinflammatory cytokines in AS pathogenesis remained unclear [38]. Macrophages derived from AS patients in response to LPS failed to stimulate the UPR but significantly upregulated IL-23 production, supporting that the IL-23/IL-17 axis is highly involved in the AS pathogenesis and is a therapeutic target [38].

(B27-HC)_2_ is a key molecule in AS pathogenesis. Most of AS patients display (B27-HC)_2_ on their PBMCs [40]. The mean grades of sacroiliitis and the lumbar spine bath ankylosing spondylitis radiology index scores are higher in AS patients with (B27-HC)_2_ on their PBMCs [40]. (B27-HC)_2_ is one of the ligands for KIR3DL2 that is present on the cell surface of NK and Th17 cells. (B27-HC)_2_ is capable of stimulating Th17 cells for IL-17 production and promoting the proinflammatory reaction. Although KIR3DL2+ cells consist of the 15% of CD4 T-cells in PBMCs, this subgroup accounts for the major numbers of Th17 in AS patients [13, 14]. A monoclonal antibody, HD6, screened from a human phage display library can specifically recognize the recombinant (B27-HC)_2_ in the binding assays [41]. HD6 can block the binding of (B27-HC)_2_ to KIR3DL2 and inhibit the survival and growth of KIR3DL2+ NK cells. HD6 suppresses the IL-17 production of PBMCs isolated from AS patients. In this study, we demonstrated that targeted delivery of the HLA-B^∗^27-binding peptide into the ER can suppress the IL-23/IL-17 axis of PBMCs isolated from AS patients (Figures 4(a) and 4(b)). Suppression of IL-17 mRNA expression in response to LPS might be due to the reduction of (B27-HC)_2_ production after treatment of PBMCs with THUC, THUA, or THUNP. Thus, stimulation of KIR3DL2+ NK and Th17 cells by (B27-HC)_2_ is decreased, diminishing the production of IL-17.

AS is a chronic inflammatory disease of the axial skeleton. The regions in joints of the spine and in the sacroiliac joint of the pelvis are mainly affected in AS patients. In serious cases, patients will gradually develop ankylosis and syndesmophytes over time [4]. Nonsteroidal anti-inflammatory drugs (NSAIDs) are regularly employed for AS treatment; TNF-*α* blockers are used in most NSAID-resistant AS patients. However, although anti-TNF-*α* therapy can eliminate much of the inflammation, development of syndesmophyte formation cannot be terminated [4, 42]. Side effects, such as an increased risk of fungal infections and of tuberculosis for the patients with latent *Mycobacterium tuberculosis* infections, are escalated after anti-TNF-*α* therapy [43], or some patients poorly respond to this therapy. In addition, the recent report has indicated that the IL-23/IL-17 axis is not affected by anti-TNF-*α* therapy in AS patients [44]. Recently, the therapeutic target of AS has been focused on the IL-23/IL-17 axis. Several potential monoclonal antibodies targeting IL-23/IL-17 pathways have been carried out in a clinical trial for AS treatment [45, 46].

## 5. Conclusion

Activation of the IL-23/IL-17 axis in AS pathogenesis is originally derived from the HLA-B^∗^27 misfolding in the ER. Our results have demonstrated that targeted delivery of the HLA-B^∗^27-binding peptide to the ER by using the THU vehicle can promote the HLA-B^∗^27 folding, decrease the (B27-HC)_2_ production, and suppress the activation of the IL-23/IL-17 axis. Our results confirmed the strong linkage between the HLA-B^∗^27 misfolding and activation of the IL-23/IL-17 axis in AS pathogenesis. Our construct for peptide delivery has a potential application in the development of the peptide therapy for AS.

## Supplementary Materials

Supplementary Materials Figure S1: Sequences of primers used for PCR.
