# Supplementary material for: Targeted Delivery of the HLA-B∗27-Binding Peptide into the Endoplasmic Reticulum Suppresses the IL-23/IL-17 Axis of Immune Cells in Spondylarthritis
Source: Mediators Inflamm. 2017 Dec 31;2017:4016802. doi: 10.1155/2017/4016802 (PMC5804395; doi:10.1155/2017/4016802)
Supplement: Supplementary Materials — Figure S1: Sequences of primers used for PCR. [file 4016802.f1.pdf]

Supplementary Figure S1: Sequences of primers used for PCR

Protein	Step	forward	reverse
THUNP	1	aggagacgacgacgaagacatcatcatcatcatcatatgcagata	ggcccagtacctgctaccacctctcagtcttaagac
	2	ggcaggaagaagaggagacgacgacgaaga	tctggtccttatggcccagtacctgctaccac
THUA	1	aggagacgacgacgaagacatcatcatcatcatcatatgcagata	ggtgaggatgcctctcttaccacctctcagtcttaagac
	2	ggcaggaagaagaggagacgacgacgaaga	gtacttcagggtgaggatgcctctctt
THU	1	aggagacgacgacgaagacatcatcatcatcatcatatgcagata	accacctctcagtcttaagacc
	2	ggcaggaagaagaggagacgacgacgaaga	accacctctcagtcttaagacc
HUA	1	catcatcatcatcatcatatgcagatattcgtgaaaaccctg	ggtgaggatgcctctcttaccacctctcagtcttaagac
	2	catcatcatcatcatcatatgcagatattcgtgaaaaccctg	gtacttcagggtgaggatgcctctctt
HUNP	1	catcatcatcatcatcatatgcagatattcgtgaaaaccctg	ggcccagtacctgctaccacctctcagtcttaagac
	2	catcatcatcatcatcatatgcagatattcgtgaaaaccctg	tctggtccttatggcccagtacctgctaccac
